# The evaluation of three treatment protocols using oral prednisone and oral meloxicam for therapy of canine idiopathic lymphoplasmacytic rhinitis: a pilot study

**DOI:** 10.1186/s13620-018-0131-3

**Published:** 2018-10-03

**Authors:** Ewa Kaczmar, Andrzej Rychlik, Marta Szweda

**Affiliations:** Department of Clinical Diagnostics, Faculty of Veterinary Medicine, University of Warmia and Mazury in Olsztyn, Oczapowskiego 14, 10-957 Olsztyn, Poland

**Keywords:** Nasal, Inflammatory, LPR, Dog, Therapy, Rhinoscopy, Glucocorticoids, Anti-inflammatories, Meloxicam, Prednisone

## Abstract

**Background:**

Idiopathic lymphoplasmacytic rhinitis (LPR) is a common inflammatory disorder of the nasal cavity in dogs due to unknown etiology. It is characterised by non-specific clinical signs, including nasal discharge, epistaxis and breathing problems. Diagnosis is usually based on the histopathologic identification of infiltrating plasmocytes and lymphocytes in the nasal mucosa and the exclusion of other underlying diseases. Treatment strategies include glucocorticoids, non-steroidal anti-inflammatory drugs, antibiotics and antifungal medications. The aim of this study was to evaluate the efficacy of various therapeutic protocols for managing canine lymphoplasmacytic rhinitis based on the results of clinical, endoscopic and histological examinations, and to determine the relapse rate for LPR in dogs.

Twenty dogs of different breeds and both sexes, aged 1 to 14 years, were divided into four groups, each consisting of five dogs, including three experimental groups diagnosed with LPR and a control group.

The dogs from the first experimental group were administered prednisone orally at 1 mg/kg/day in the first 4 weeks and 0,5 mg/kg/day in the following 2 weeks. The second group of dogs was administered meloxicam orally at 0,1 mg/kg/day in the first 3 weeks, followed by prednisone at 1 mg/kg/day in the following 2 weeks and 0,5 mg/kg/day in the last week of the treatment. The dogs from the third experimental group were administered meloxicam orally at 0,1 mg/kg/day for 6 weeks. The control group of dogs was administered empty gelatin capsules (placebo) orally for 6 weeks. Clinical signs, endoscopic and histopathologic lesions were scored before and after treatment. Groups were compared using Chi- squared statistics in a 2 × 2 table for pre- versus post-treatment scores.

**Results:**

Clinical signs persisted in the group treated with meloxicam and were mostly resolved in prednisone-treated dogs. However, endoscopic and histological changes were still observed in these two groups after treatment. The severity of all diagnostic features was reduced in the group treated with meloxicam for 3 weeks followed by prednisone for 3 weeks. The significant differences (*p* < 0.05) were noted between experimental and control groups. The dogs showed a statistically significant reduction in characteristics of the LPR before and after treatment, as measured by clinical signs (Group 1vs.4 *p* = 0.00, group 2 vs 4 *p* = 0.00, group 3 vs 4 p = 0,01), by endoscopy (1 vs 4 *p* = 0,01, 2 vs 4 *p* = 0,00, 3 vs 4 *p* = 0,03), and by histopathology (groups 1 vs 4 p = 0,00, 2 vs 4 p = 0,00, 3 vs 4 p = 0,03). The significant differences were noted between experimental groups, as measured by endoscopy (group 2vs 3 *p* = 0,04), and by relapse rate (groups 1 and 2 p = 0,03, groups 2 and 3 p = 0,01).

**Conclusions:**

The three treatment protocols administered to dogs improved clinical, endoscopic and histological status. However, oral administration of meloxicam for 3 weeks, followed by prednisone for 3 weeks, appeared to be the most successful treatment. These patients remained asymptomatic for 6 months.

## Background

Previous studies have shown that idiopathic lymphoplasmacytic rhinitis (LPR) is the second most common chronic disease of the nasal cavity in dogs, after nasal neoplasia [1–4]. Rhinitis caused by foreign bodies or secondary to odontogenic disorder is less frequently diagnosed in dogs [1, 4].

The clinical features of LPR include nasal discharge, epistaxis, reverse sneezing, stridor and breathing problems, such as inspiratory dyspnea [1, 2, 4–6]. These signs are not considered pathognomonic, as they may be related to various diseases of the upper respiratory tract [3–5, 7, 8]. Most dogs with LPR present unilateral or bilateral nasal discharge during the clinical examination. However, previous studies suggest that LPR is most often a bilateral disease [7]. The type of nasal discharge could be serous, mucoid, mucopurulent or haemorrhagic at presentation [4, 5]. While certain studies show that the disease rarely provides characteristic radiographic evidence, other studies prove that LPR is associated with marked radiographic lesions, such as nasal turbinate destruction [1, 4, 8]. Advanced diagnostic modality such as computed tomography (CT) is considered superior to radiography for the diagnosis of LPR [2, 9]. Computed tomography defines the extend and severity of abnormalities of the nasal cavity [9]. Turbinate destruction, fluid accumulation, soft tissue opacification, gas pocketing, and frontal sinus involvement are common CT findings in course of this condition [2, 7]. However, magnetic resonance imaging (MRI) as another previously described modality is providing superior soft- tissue detail compared with CT, and could aid in the differentiation of sino-nasal aspergillosis (SNA) and LPR in dogs [2, 10]. Hyperaemic nasal mucosa and accumulation of mucous discharge are observed during rhinoscopy [3, 4]. Non-pathogenic bacterial and fungal cultures are found in most cases of idiopathic LPR and represent normal nasal flora or secondary infection rather than presence of pathogenic bacteria and primary infection [2, 4, 8]. In pathology reports, LPR is characterised by the presence of lymphocytes and plasmocytes infiltrating the nasal mucosa [2–4]. Finally, the definitive diagnosis of idiopathic LPR is made by the exclusion of other causes of nasal disease [2, 10].

The aetiology of LPR still remains unknown. Previous studies revealed that innate immunity and hypersensitivity could be implicated in the pathogenesis of LPR [1–3, 8, 11, 12]. Odontogenic infections could also be involved in LPR [13]. According to some studies LPR is a complex disorder that could arise in consequence of bacterial and fungal infections [2]. It has also been hypothesised that LPR represents undiagnosed neoplastic disease or sino-nasal aspergillosis (SNA) [2]. Although higher levels of fungal DNA have been demonstrated in nasal tissue from dogs with LPR compared to dogs with neoplasia [14], cytokine and chemokine expression in SNA and LPR is different, constituting two distinct entities [2, 15].

Idiopathic lymphoplasmacytic rhinitis proves to be not only diagnostic but also a therapeutic challenge for clinicians. There are no specific protocols for treating the disease, and clinical trials are lacking. Partial to almost complete responses to glucocorticoids have been reported [2, 4, 10]. Anti-inflammatory drugs and antibiotics have shown various therapeutic effects [2, 4]. Treatment strategies also include antifungal medications and inhalant steroids [2, 6]. One study compared the efficacy of anti-inflammatory dose of prednisone with a cyclosporine and desensitisation therapy [4]. Other studies show that an immunosuppressive dose of prednisone or other immunosuppressive drugs could also be effective [2, 9]. However, the effectiveness of meloxicam monotherapy and meloxicam in sequence with prednisone has not been investigated. Furthermore, to the author’s knowledge there is no information available concerning the relapse rates for LPR after such therapies.

The aim of this study was to evaluate the efficacy of three different therapeutic protocols for managing canine lymphoplasmacytic rhinitis based on the results of clinical, endoscopic and histological examinations, and to determine the relapse rate for LPR in dogs.

## Methods

The study was performed on 20 dogs which were admitted to the Veterinary Clinic of the Faculty of Veterinary Medicine at the University of Warmia and Mazury in Olsztyn and diagnosed with LPR from October 2016 to October 2017. All procedures were approved by the Local Ethics Committee for Animal Experimentation in Olsztyn (No. 47/2009/DTN). All clients gave written informed consent. Investigators were unaware of the treatment assignment. This was a randomized, controlled and prospective study with stratified sampling.

The dogs were enrolled for the experiment based on a signalment, history and the results of a clinical examination, a radiographic examination of the nasal cavity, bacteriological and mycological analyses of nasal swabs, a rhinoscopic evaluation and a histological analysis of nasal biopsy specimens. Furthermore, none of the dogs could show evidence of other chronic disease (kidney, liver, GI tract) based on results obtained from initial diagnostic testing nor could have received corticosteroids or NSAIDs. Nasal discharge and other clinical signs, such as stridor, were evaluated during a clinical examination. Radiography of the nasal cavities included dorsoventral intraoral view and rostrocaudal view of frontal sinus. Specimens for bacteriologic examination were processed by using standard culture technique. Samples for mycologic studies were submitted both on the agar-gel double immunodifussion and enzyme-linked immunosorbent assay (ELISA). According to previous studies confirmation of fungal disease required at least three positive ancillary diagnostic tests (radiographic, endoscopic, mycologic, histopathologic) [10]. The rhinoscopy was performed in all dogs with a double endoscopic technique: a retrograde approach and anterograde approach. The nasopharynx was examined using the flexible endoscope (Olympus URF-P5, Olympus, Japan) inserted orally (retrograde approach) with a diameter of 3 mm and a length of 70 cm. Both nostrils (the rostral part until the ethmoidal volutes of the nasal cavity- the anterograde approach) were examined with the use of a rigid endoscope (Karl Storz, Germany) with a diameter of 2.7 mm with a 30^0^ angle and a length of 19 cm. During rhinoscopy, nasal mucosa was subjected to an endoscopic evaluation, and 3 or 4 biopsy specimens were collected for histological analyses before and after treatment. A 2,5 mm endoscopic forceps was used for biopsy. In addition, in all dogs nasal flush was performed during rhinoscopy to allow appropriate visualisation and to exclude potential foreign body [5]. Biopsy specimens were fixed in 10% buffered formalin, embedded in paraffin, sectioned and stained with hematoxylin and eosin. All the slides were reviewed blindly by the same pathologist. Finally, lymphoplasmacytic rhinitis was diagnosed based on the presence of infiltrating plasmocytes and lymphocytes in mucosal specimens in the histological analysis and the exclusion of other underlying diseases [2, 10].

The dogs were divided into four treatment groups of 5 dogs each. The first group was administered prednisone (Encorton, Polfa Pabianice S. A., Poland) orally at 1 mg/kg/day in the first 4 weeks and 0.5 mg/kg/day in the following 2 weeks. The second group was administered meloxicam (Gromeloksin, Biowet Drwalew, Poland) orally, a non-steroidal anti-inflammatory drug, at 0,1 mg/kg/day in the first 3 weeks, followed by prednisone (Encorton, Polfa Pabianice S. A., Poland) at 1 mg/kg/day in the following 2 weeks and 0.5 mg/kg/day in the last week of the study. The third group was administered meloxicam (Gromeloksin, Biowet Drwalew) orally at 0,1 mg/kg/day for 6 weeks. The fourth group was administered placebo (empty gelatin capsule) orally once daily for 6 weeks. In order to reduce incidence of potential adverse events related to both meloxicam and prednisone or prednisone/ meloxicam alone, these three groups of dogs mentioned, were also administered pantoprazole (Controloc, Takeda Pharma) orally at 2 mg/kg/day in the first 3 weeks and 1 mg/kg/day in the following 2 weeks, and 0,5 mg/kg/day in the last week of the study. Dosages and duration of using prednisone and meloxicam were selected on the basis of published recommendations [16–21]. Administration of pantoprazole (2 mg/kg/day) was also selected on the basis of published recommendations [20–22]*.* The dosage of pantoprazole was tapered to the lowest effective dose before discontinuation on the basis of information extrapolated from human studies and then generated in dogs [23–25]. The owners were instructed to notify the attending veterinarian if their dog was vomiting, lethargic, polyuric, polydipsic or having diarrhea or melena.The examined population consisted of 13 males and 7 females, and it was represented by the following breeds: mixed-breed dogs (8/20), German Shepherds (4/20), Dachshunds (3/20), Yorkshire Terriers (3/20), Labrador Retrievers (2/20). The age of dogs in groups ranged from 1,5 to 14 years (mean 5,9 years). The groups were similar in relation to age, breed, sex and time of the year.

Efficacy of the treatments was assessed by a clinical, endoscopic and histopathological pre- and post-treatment scores each calculated by the results of a physical examination, rhinoscopy and histopathology, as appropriate. The clinical scoring system evaluated the following items: composition of discharge, frequency and intensity of discharge, and frequency and intensity of associated clinical signs (nasal stridor, sneezing etc.). Composition of discharge was classified as serous/ sero-mucous, mucous/ sero-purulent, purulent/ muco-purulent/ haemorrhagic (scale 0 [absent] to 3 [purulent/ muco-purulent/haemorrhagic], see Table 1, composition of discharge). The frequency and intensity of discharge were assessed as rare (< 2 times/week), frequent (> 2 times/week but intermittently) and continuous (without interruption), and mild, moderate and severe, respectively [5]. This was also converted into numerical score 0–3 (see Table 1, frequency and intensity of discharge). Third item of the clinical score was frequency and intensity of other clinical signs classified as mild, moderate and severe according to criteria given above (see Table 1, frequency and intensity of other clinical signs). The overall score ranged from 0 to 9, with clinical signs of LPR interpreted as clinically insignificant/absent (score 0–2, dog assigned as -), mild (score 3–5, dog assigned as +), moderate (score 6–8, dog assigned ++), or severe (9 points, dog assigned as +++). This system was mostly created based on previous described characteristics of canine nasal discharge by Plickert et al. [5]. Analogous to the clinical scoring system, endoscopic scoring system was created and calculated (see Table 2, specific criteria for assessment of endoscopic findings). This endoscopic scoring system evaluates the following items: mucous accumulation, lesions of hyperaemia, and turbinate oedema. These were selected according to previous described cumulative rhinoscopic score by Johnson et al. [26]. Histopathology was assessed based on histologic nasal inflammation scoring system in dogs with inflammatory nasal disease proposed by Furtado et al. [27] (see Table 3, specific criteria for assessment of histopathological changes). In this system, the proportion of each inflammatory cell type (lymphocytes, neutrophils, plasma cells and eosinophils), severity of inflammation, ephitelial and goblet cell hyperplasia, oedema presence were each graded as mild, moderate, or severe [27]. The dogs’ condition was monitored for up to 6 months after therapy to determine the relapse rate for canine LPR. Follow-up information was obtained by re-examination and by telephone contact with the attending clinician or dogs owners. Therefore, the relapse rate classification was established based on clinical, rhinoscopy and histopathology scores 6 months after cessation of treatment. The overall score for relapse rate ranged from 0 to 9, with clinical, or endoscopic or histopathology score of LPR interpreted as clinically insignificant/absent (score of 0 points, dog assigned as -), mild (score 1–3 points, dog assigned as +), moderate (score of 4–6 points, dog assigned ++), or severe (7–9 points, dog assigned as +++). The relapse rate mean for each treatment group was assessed based on cumulative clinical, endoscopic and histological scores after 6 months in comparison to scores before treatment. Results reported as percent, referred to mean change of numbers of + (i.e + as 1, ++ as 2, +++ as 3) scored as specific criteria +, ++, +++ (see Table 5, relapse rate classification).

**Table 1:** Specific criteria for assessment of clinical efficacy parameters and scoring system

Clinical examination parameter	Description	Score	Final overall score of clinical signs
Composition of discharge	Absent	0	0–2 (−) absent 3–5 (+) mild 6–9 (++) moderate 9 (+++) severe
	Serous/ sero-mucous	1	
	Mucous/ sero- purulent	2	
	Purulent/ muco-purulent/ haemorrhagic	3	
Frequency and intensity of discharge	Absent	0	
	Rare < 2 times/week, mild	1	
	Frequent > 2 times/week, moderate	2	
	Continuous, severe	3	
Other clinical signs (nasal stridor, reverse sneezing etc)	Absent	0	
	Rare < 2 times/week, mild	1	
	Frequent > 2 times/week, moderate	2	
	Continuous, severe	3

**Table 2:** Specific criteria for assessment of endoscopic efficacy parameters and scoring system

Parameter	Description	Score	Final overall score of endoscopic changes
Mucous accumulation	Absent	0	0–2 (−) absent 3–5 (+) mild 6–9 (++) moderate 9 (+++) severe
	Serous/ sero-mucous, small amount- mild	1	
	Mucous/ sero- purulent, small amount- moderate	2	
	Purulent/ muco-purulent/ haemorrhagic, large amount- severe	3	
Severity of hyperaemia	Absent/ normal mucosa	0	
	Mild (present in less than 20% of nasal cavity)	1	
	Moderate (20–40% of nasal cavity)	2	
	Severe (> 40% of nasal cavity)	3	
Turbinate oedema	Absent/normal mucosa	0	
	Oedematous mucosa	1	
	Marked oedema	2	
	Polypoid mucosa	3

**Table 3:** Specific criteria for assessment of histopathology and scoring system

Parameter	Description	Score	Final overall score of histopathology
Neutrophils	Not present	0	(0–12)
Lymphocytes	100–200 cells per 3 fields- mild	1	
Plasma cells	201–300 cells per 3 fields- moderate	2	
Eosinophils	> 300 cells per 3fields- severe	3	
Total inflammation score (0–12)	Mild (0–3)	1	0–2 (−) absent 3–5 (+) mild 6–9 (++) moderate 9 (+++) severe
	Moderate (4–6)	2	
	Severe > 7	3	
Epithelial and goblet cell hyperplasia	Mild (focal thickened epithelium)	1	
	Moderate (multifocal thickened epithelium)	2	
	Severe (diffuse thickened epithelium)	3	
Mucosal oedema	Mild (present in less than 20% of 3 random fields)	1	
	Moderate (20–40% of 3 random fields)	2	
	Severe (> 40% of 3 random fields)	3	

Statistical analysis was carried out with Statistica 6.0. (Stat Soft Inc., Tulsa, OK, USA). Treatment groups were evaluated for the differences at baseline with Wilcoxon’s nonparametric tests. Categoric variables were compared using Chi- squared statistics with the data arranged in a 2 × 2 table for pre- versus post-treatment scores. For all tests, *p* values *p* < 0.05 were considered significant.

## Results

A clinical examination revealed persistent, bilateral mucous and serous discharge or serous and purulent discharge in all dogs, with bloody discharge in 3 cases. Pathological changes were not observed in a radiological examination, such as mass, turbinate destruction, frontal sinus involvement. However, slight to no opacification was observed. Therefore, nasal radiographs were considered unremarkable in all cases.

During rhinoscopy performed before treatment, small amounts of mucous and serous discharge from both nostrils were observed in 12 dogs (1A, 1B, 1C; 2A, 2B, 2C; 3A, 3B, 3C, 4A, 4B, 4C), and serous and purulent discharge– in 8 dogs (1D, 1E; 2D, 2E; 3D, 3E, 4D, 4E). In addition, the presence of blood was recorded in nasal discharge from 3 dogs (one from each group- 1A; 2A; 3A). In the rostral part of the nasal cavity, mucosal hyperaemia was recorded in the ventral concha and in basal (plica basalis), alar (plica alaris) and straight (plica recta) folds in all dogs. The nasal conchae were markedly oedematous in 2 dogs from each group (1A, 1B; 2A, 2B; 3A, 3B, 4A, 4B). Inflammatory polyps confirmed by histopathology were detected in ventral nasal meatuses of 3 German Shepherds (1A, 2A, 3A– one from each group). However, these lesions were not observed in a German Shepherd from the third group (3B).

Bacteriological and mycological assays did not reveal the presence of pathological bacteria or fungi in any of the evaluated dogs.

A histological examination of biopsy specimens revealed lymphocytic and neutrophilic mucosal exocytosis before therapy. The epithelium was hyperplastic and infiltrated by lymphocytes and plasma cells. Lamina propria showed vascular proliferation and fibroplasias.

In the first group of dogs, the applied treatment eliminated nasal discharge in 4 dogs after 7 to 10 days duration of the therapy, but mild serous discharge persisted in the German Shepherd (1A) (see Table 4, Group 1, clinical signs after treatment). In the second group, clinical items scored were resolved during first week in all five dogs (see Table 4, Group 2, clinical signs after treatment). In the third group, moderate signs of LPR, such as serous nasal discharge were observed in one dog (3A) which had been diagnosed with severe clinical signs, such as haemorrhagic nasal discharge before therapy. The remaining dogs showed mild clinical signs scored (3B, 3C) or no signs of LPR (3D, 3E) (see Table 4, Group 3, clinical signs after treatment). In the fourth group of dogs, the severity of nasal discharge increased after 6 weeks of placebo administration (see Table 4, Group 4, clinical signs after treatment).

**Table 4:** The results of clinical, endoscopic and histopathological examinations conducted before and after treatment

		Before treatment			After treatment		
Group	Dog	Clinical signs	Endoscopic changes	Histopathological changes	Clinical signs	Endoscopic changes	Histopathological changes
1	A	+++	+++	++	+	++	+
	B	++	++	+	–	+	+
	C	+	+	+	–	–	–
	D	+	+	+	–	–	–
	E	++	++	++	–	+	+
2	A	+++	+++	+++	–	–	+
	B	++	+	++	–	–	+
	C	++	++	++	–	–	–
	D	+	+	++	–	–	–
	E	++	+	++	–	–	–
3	A	+++	+++	+++	++	++	+
	B	++	++	++	+	+	++
	C	++	++	++	+	+	–
	D	+	+	++	–	–	+
	E	+	++	+	–	+	–
4	A	++	++	++	+++	++	++
	B	++	+	+	++	++	++
	C	++	++	++	+++	++	+++
	D	+	+	+	++	+	++
	E	+	+	++	++	++	++

- no changes (score of 3–5 points)

+ mild signs/changes (score of 3–5 points)

++ moderate signs/changes (score of 6–8 points)

+++ severe signs/changes (score of 9 points)

Group 1 - prednisone (Encorton)

Group 2- meloxicam (Gromeloksin), followed by prednisone (Encorton)

Group 3- meloxicam (Gromeloksin)

Group 4- placebo (empty gelatin capsules)

**Fig. 1:**
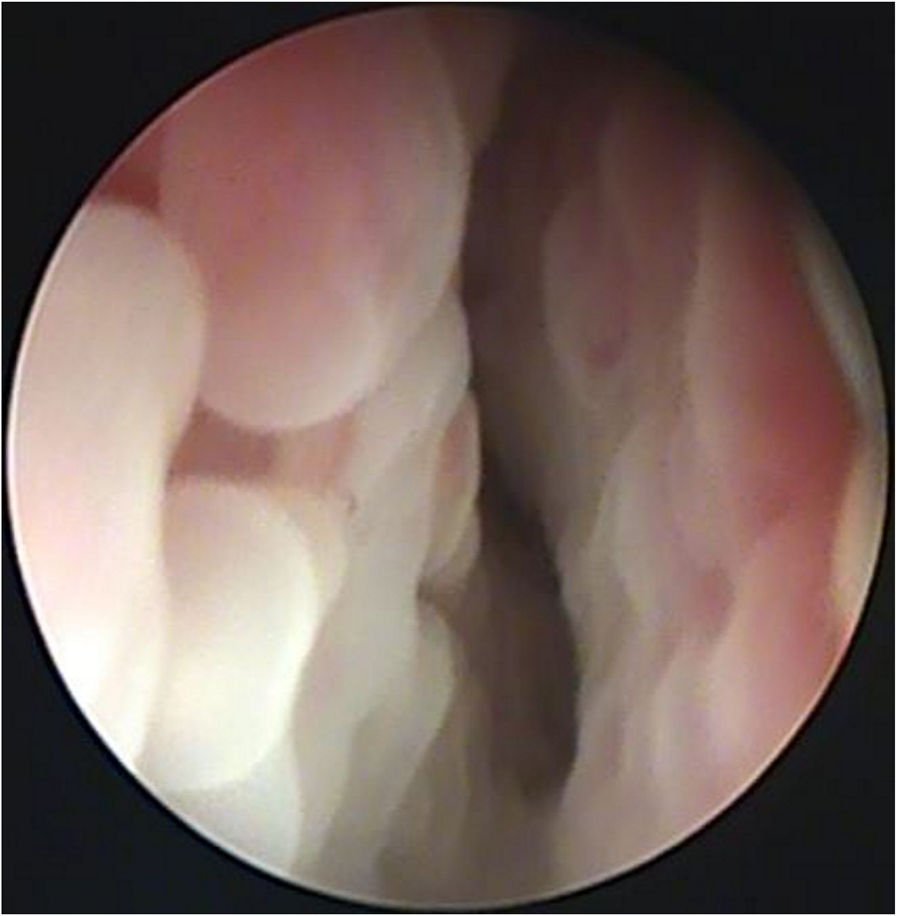
Inflammation observed in the first rhinoscopic evaluation of a 7-year-old German Shepherd (2A, Group 2, Table 4, Before treatment, Endoscopic changes) subjected to combination therapy; assigned as +++ as severe endoscopic changes in total endoscopic scoring system (Table 2)

**Fig. 2:**
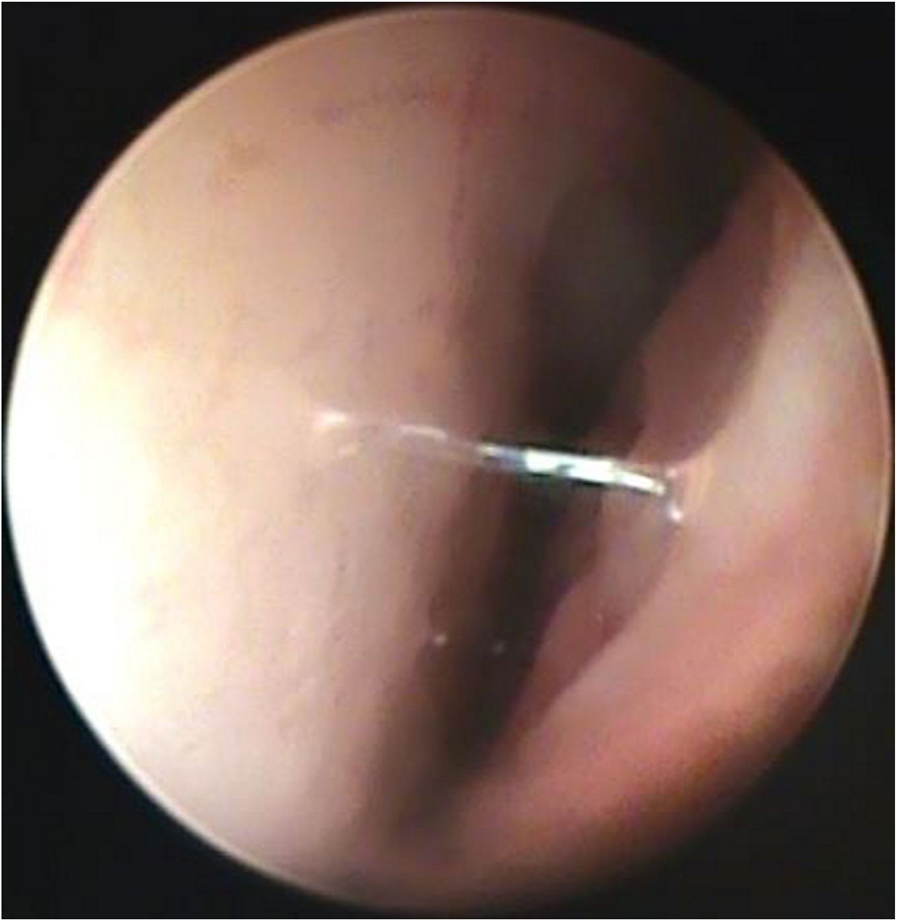
Absence of changes in the same German Shepherd (2A, Group 2, Table 4, After treatment, Endoscopic changes) after combination therapy assigned as –, as no endoscopic changes in total endoscopic system

In the first group, an endoscopic evaluation after treatment revealed moderate accumulation of serous discharge (1A) or no discharge with reduced hyperaemia of mucosal folds (1B, 1E). In another two cases (1A, 1B), the applied treatment alleviated turbinate oedema. In the German Shepherd (1A), polyps in the ventral nasal meatus were eliminated in the proximal segment, but were still visible in distal segments (see Table 4, Group 1, endoscopic changes after treatment). In the second group, nasal discharge and mucosal hyperaemia were not observed in any of the dogs. Turbinate oedema was eliminated in another 2 dogs (2A, 2B) (see Table 4, Group 2, endoscopic changes after treatment). The treatment resolved inflammatory polyps in the German Shepherd (2A) (see Figs. 1, 2). In the third group, nasal discharge and mucosal hyperaemia were moderate in one dog (3A) and less pronounced in 3 other cases (3B, 3C, 3E) after therapy. Turbinate oedema was eliminated in one dog (3B), but persisted in another dog (3A). In the German Shepherd (3A), polyps were observed only in the distal segment of the nasal meatus (see Table 4, Group 3, endoscopic changes after treatment). In the fourth group, nasal discharge and turbinate oedema were severe in all five dogs. (4A, 4B, 4C, 4D, 4E) (see Table 4, Group 4, endoscopic changes after treatment).

After treatment, a histological analysis revealed mild (1A, 1B, 1E) or no features of LPR in the first group (see Table 4, Group 1, histopathological changes). In the second group, mild histological changes were observed in 2 dogs (2A, 2B). These changes were considered as an improvement from severe (2A) and moderate (2B) in comparison to pre-treatment histopathology (Table 1, Group 2, histopathological changes). In the third group, the severity of histological changes was not reduced in one dog (3B), and mild changes in another two cases (3A, 3D) or no changes were noted in the remaining dogs (see Table 4, Group 3, histopathological changes). In the fourth group, severe histological changes were observed in all five dogs. (4A, 4B, 4C, 4D, 4E) (see Table 4, Group 4, histopathological changes). The results of clinical, endoscopic and histological examinations conducted before and after treatment are summarised in Table 1.

The relapse rates were estimated at 75% in dogs treated with NSAID and 50% in dogs administered a glucocorticoid. The dogs subjected to combination therapy remained asymptomatic 6 months after treatment. Therefore, relapse rate scored at 0% (see Table 5, Relapse rate classification). Results reported as percent, referred to the mean change of numbers of + (i.e + as 1, ++ as 2, +++ as 3, +/− as 0,5) scored as specific criteria +, ++, +++. In relapse rate classification, one dog (1D) was assigned as +/−. According to clinical score system presented, this dog was assigned as – after clinical examination. However, the owner reported that reverse sneezing occured incidentally. Therefore, in this particular case the clinical signs was assigned as +/−, as uncertain.

**Table 5:** Relapse rate classification. Comparison of the results of clinical, endoscopic and histopathological examinations conducted before treatment and after 6 months

		Before treatment			6 months after treatment			Relapse rate%
Group	Dog	Clinical signs	Endoscopic changes	Histopathological changes	Clinical signs	Endoscopic changes	Histopathological changes	
1	A	+++	+++	++	+	+	++	(12,5/25) 50
	B	++	++	+	+	+	+	
	C	+	+	+	–	+	+	
	D	+	+	+	+/−	–	–	
	E	++	++	++	+	+	+	
Total	25				12,5			
2	A	+++	+++	+++	–	–	–	(0/29) 0
	B	++	+	+	–	–	–	
	C	++	++	++	–	–	–	
	D	+	+	++	–	–	–	
	E	++	+	++	–	–	–	
Total	29				0			
3	A	+++	+++	+++	++	++	+++	(22/29) 75
	B	++	++	++	+	+	++	
	C	++	++	++	+	+	++	
	D	+	+	++	+	+	+	
	E	+	++	+	+	+	++	
Total	29				22			

- no changes (score of 0 points)

+/− unremarkable

+ mild signs/changes (score of 1–3 points)

++ moderate signs/changes (score of 4–6 points)

+++ severe signs/changes (score of 7–9 points)

Group 1 - prednisone (Encorton)

Group 2- meloxicam (Gromeloksin), followed by prednisone (Encorton)

Group 3- meloxicam (Gromeloksin)

Group 4- placebo (empty gelatin capsules)

The dogs from the control group after the duration of this study were given treatment which was the most effective. Therefore, they were not included in relapse rate classification.

Treatment groups were evaluated for the differences at baseline with Wilcoxon’s nonparametric tests (see Table 6, Wilcoxon’s analysis). The significant differences (*p* < 0.05) were found between experimental (1, 2, 3) and the control (4) groups. The dogs showed a statistically significant reduction in characteristics of LPR before and after treatment, as measured by clinical signs (see Table 7, Differences between pre- and post-treatment scores for clinical signs, Groups 1–4, 2–4, 3–4), by endoscopy (see Table 8, Differences between pre- and post-treatment scores for endoscopic changes, Groups 1–4, 2–4, 3–4), and by histopathology (see Table 9, Differences between pre- and post-treatment scores for histopathological changes, Groups 1–4, 2–4, 3–4). The significant differences were noted between experimental groups, as measured by endoscopy (see Table 8, Differences between pre- and post-treatment scores for endoscopic changes, Groups 2–3), and by relapse rate (see Table 10, differences between pre- and post-treatment scores for relapse rate, Groups 1–2, 2–3). The remaining results of comparisons between experimental groups were insignificant (see Table 7, Table 8, Table 9, Table 10).

**Table 6:** Wilcoxon’s analysis within groups

Group	*P* value
1	0,04
2	0,04
3	0,04
4	0,04

Group 1 - prednisone (Encorton)

Group 2- meloxicam (Gromeloksin), followed by prednisone (Encorton)

Group 3- meloxicam (Gromeloksin)

Group 4- placebo (empty gelatin capsules)

**Table 7:** Differences between pre- and post-treatment scores for clinical signs

Groups	*P* value
1–2	0,19
1–3	0,23
2–3	0,08
1–4	0,00
2–4	0,00
3–4	0,01

Group 1 - prednisone (Encorton)

Group 2- meloxicam (Gromeloksin), followed by prednisone (Encorton)

Group 3- meloxicam (Gromeloksin)

Group 4- placebo (empty gelatin capsules)

**Table 8:** Differences between pre- and post-treatment scores for endoscopic changes

Groups	*P* value
1–2	0,08
1–3	0,37
2–3	0,04
1–4	0,01
2–4	0,00
3–4	0,03

Group 1 - prednisone (Encorton)

Group 2- meloxicam (Gromeloksin), followed by prednisone (Encorton)

Group 3- meloxicam (Gromeloksin)

Group 4- placebo (empty gelatin capsules)

**Table 9:** Differences between pre- and post-treatment scores for histopathological changes

Groups	*P* value
1–2	0,58
1–3	0,18
2–3	0,15
1–4	0,00
2–4	0,00
3–4	0,03

Group 1 - prednisone (Encorton)

Group 2- meloxicam (Gromeloksin), followed by prednisone (Encorton)

Group 3- meloxicam (Gromeloksin)

Group 4- placebo (empty gelatin capsules)

**Table 10:** Differences between pre- and post-treatment scores for relapse

Groups	*P* value
1–2	0,03
1–3	0,12
2–3	0,01

Group 1 - prednisone (Encorton)

Group 2- meloxicam (Gromeloksin), followed by prednisone (Encorton)

Group 3- meloxicam (Gromeloksin)

## Discussion

The published data indicate that LPR is one of the main causes of persistent nasal disease in dogs [1, 2]. The aetiology and pathogenesis of LPR are unknown, as well as how to treat this condition. Previous studies have shown that allergies and immune disorders could be involved in the disease [1–3, 8, 11, 12]. It has been suggested that LPR was associated with the presence of foreign objects, undiagnosed tumors or fungal infections in the nasal cavity [2]. However, the involvement of bacterial and fungal infections in the pathogenesis of LPR is only hypothetical. Mercier reported that LPR is not associated with pathogenic bacteria or fungi [6], whereas Windsor showed that LPR is a complex disorder that could arise in consequence of bacterial and fungal infections [2]. In addition, Peeters et al. demonstrated that cytokine and chemokine expression profiles in SNA and LPR differ, implying two different disease entities [15]. Odontogenic infections could also be implicated in LPR [13].

In our study, the most effective treatment led to complete resolution in some dogs, whereas mild histological changes persisted in other patients (2A, 2B), which suggests that LPR has a complex etiology [2]. Moreover, the severity of signs and changes after 6 weeks of placebo administration suggests, that LPR has progressive character (Table 1, Group 4, After treatment).

Inflammation of nasal mucosa are most frequently diagnosed in middle-aged dogs (mean 9 years) ranged from 2.3–17 years [4] or 1.5–14 years of age (mean 8.5 years) [2, 7]. The results of our study are not consistent with the findings mentioned above. In our study, the dogs age ranked from 1 to 14 years (mean 5,9 years). The majority of the evaluated patients were male (13/20). However, there are no reports to indicate that the incidence of LPR is higher in either gender. Some studies showed that Yorkshire Terriers, Dachshunds and German Shepherds could be more predisposed to the disease [3, 4]. German Shepherds were also one of the most prevalent breed in our study (4/20). In this experiment, bilateral nasal discharge was noted in all dogs. Windsor et al. revealed that bilateral nasal discharge is indicative of LPR because tumors and foreign bodies in the nasal cavity are generally associated with unilateral discharge, at least in the initial stage of disease [7].

The radiographic findings were considered unremarkable. This results are consistent with previous studies suggested that slight to moderate increase in radiographic opacity, or no opacification, or absence of radiographic lesions could occur in dogs with LPR [1, 3, 4, 8]. Radiographic signs of LPR demonstrate a spectrum of appearances ranging from minor in-creases in soft tissue opacity to lytic bone lesions [3]. However, destructive pattern is suggestive of fungal infection or neoplasia [1–3]. Previous studies described that in most cases radiographic signs of LPR included increase in opacity, turbinate destruction, or normal frontal sinus [1–3]. In present study, unremarkable radiographic findings may suggesting the early diagnosis of LPR or similar stage of the disease in most dogs.

In this study samples for mycologic studies were submitted both on the agar-gel double immunodifussion and enzyme-linked immunosorbent assay (ELISA). However, because of its sensitivity, serology is not considered a good screening test for SNA in dogs suffering from chronic nasal discharge [28]. According to previous studies confirmation of fungal disease required at least three positive ancillary diagnostic tests (radiographic, endoscopic, mycologic, histopathologic) [10].

In this study, it is documented that the therapy combination with a non-steroidal anti-inflammatory drug and a glucocorticoid (group 2) was most effective in resolving the clinical signs of LPR. It is important to note that glucocorticoids should not be given concurrently with NSAIDs or with caution, due to the increased risk of gastrointestinal ulceration [17]. Particular care should be taken when considering sequential NSAID and glucocorticoid therapy [17]. Gastroprotective drugs may be indicated prophylactically [17, 21]. The dogs with combined treatment showed no gastrointestinal signs before and after the therapy. No clinically significant adverse events occured in association with meloxicam and prednisone administration. A specific protocol of pantoprazole with tapering dosage was introduced to prevent the rebound acid hypersecretion and hypergastrinemia in dogs [25]. This phenomenom has been evidenced in humans [29, 30]. However, the clinical significance in veterinary patients is still unknown [30].

There are limited studies on the use of PPIs in dogs. However, in one study, omeprazole decreased the degree of gastritis associated with aspirin therapy, whereas cimetidine (H2 antagonist) did not [24]. In a study evaluating the efficacy of PPIs in reducing the incidence of gastric lesions in dogs receiving corticosteroids, a trend toward improvement in mucosal lesions was noted [24]. There is also a gastric cytoprotective agent with both acid-inhibitory and mucosal-protective properties, such as misoprostol. The primary indication for misoprostol is prevention of NSAID-induced ulcers [22, 24]. However, misoprostol is not particulary effective in healing existing NSAID-induced ulcers in comparison with PPIs [21, 22, 24]. Furthermore, it is not clearly effective in protecting dogs receiving NSAIDs as has been reported in human medicine [22]. Two separate studies have shown that misoprostol administration had no effect on the incidence of gastric haemorrhage [24]. Moreover, misoprostol has a short half-life and must be given two to three times daily [22]. Therefore, its greater cost, need for frequent administration, and higher rate of adverse effects (i.e. diarrhea, abortifacient effect) suggest that PPIs may be preferable [21, 22].

It is also worth considering that meloxicam as a selective inhibitor of COX-2, is a drug whose therapeutic effects are as strong as conventional NSAIDs, such as piroxicam suggested in treatment of LPR in previous studies [2], but which leads to fewer side effects [31, 32]. Meloxicam is well tolerated by dogs [33]. It has been as effective as other NSAIDs and was shown to have better safety profile [33]. Therefore, it may be preferable in long-term use rather than piroxicam [32]. Moreover, some studies suggested that meloxicam may have immunosuppressive effects and ability to inhibit lymphocyte proliferation [34]. Therefore, this could have applications in anti-inflammatory therapy [34–36]. In present study, we hypothesised that the group treated with meloxicam followed by prednisone may present better clinical effects than with prednisone or meloxicam alone. In our daily practice, we have observed good, but unsatisfactory outcomes in some meloxicam-treated dogs during the 3-week treatment period. Therefore, we introduced prednisone, and interestingly, the response was complete after another 2- or 3-week treatment period. Switching this these classes of drugs occurs in different chronic diseases, such as i.e. osteoarthritis, inflammatory bowel disease (IBD). Medical treatment for induction of clinical remission of IBD is largely empirical and consists of use of anti-inflammatory drugs, with corticosteroids providing the most consistent benefit [37]. For instance, clinical trials in human IBD consistently demonstrate response to 5-aminosalicylates and corticosteroids as mainstays of treatment [37]. Further studies are needed to clarify all the effects and abilities of NSAIDs and SAIDs in therapy of LPR.

In present study nasal discharge persisted in the group treated with NSAID alone. According to information from the client during the 6 weeks of initial therapy nasal discharge was quickly resolved in dogs administered a glucocorticoid, but endoscopic and histological changes were still observed in this group after treatment. In the group treated with placebo, the severity of clinical signs, endoscopic and histological changes significantly increased. Relapse rates are a robust indicator of response to the treatment in persistent diseases. In the present study, relapse rates were estimated at 75% in dogs treated with NSAID and 50% in dogs administered a glucocorticoid. The dogs subjected to combination therapy remained asymptomatic 6 months after treatment.

Lobetti compared the efficacy of a starting dose of prednisone at 1 mg/kg, administered per os once daily for 7–10 days, followed by 0.5 mg/kg, with a cyclosporine dose of 5 mg/kg, administered per os once daily for minimum 4 weeks, and desensitisation therapy. The latter treatment was most effective, and it eliminated the symptoms of disease in all dogs [4]. Lobetti and Windsor concluded that glucocorticoids lead to relapse [4, 7], whereas Burgener reported that glucocorticoids effectively resolved the signs of LPR in 4 out of 5 dogs, i.e. in 80% of the dogs [12]. The therapy combination with NSAIDs and SAIDs has never been studied in dogs with LPR, and it proved to be the most effective treatment in our study. Antibiotics are not effective, but they can induce a temporary improvement in the patient’s condition by eliminating signs of secondary bacterial infection, such as serous and purulent nasal discharge [2, 7, 13]. A recent study by Lappin recommends administration of doxycycline for both nasal and respiratory co-infections (*Mycoplasma spp, B. bronchiseptica* etc) [38]. In addition, the effectiveness of drugs of this class for the treatment of LPR may be attributed partially to its anti-inflammatory or immunomodulating effects [4].

In this study, the effectiveness of steroids, administered both alone and in combination with other drugs, could support the hypothesis that LPR has immune or allergic etiology. The glucocorticoid was administered in an anti-inflammatory rather than an immunosuppressive dose, and a similar treatment protocol was described by Lobetti [4]. However, a study by Van Pelt shows that an immunosuppressive dose of prednisone at 2 mg/kg was effective in 90% of the evaluated animals [11]. Additionally, it should be noted that higher glucocorticosteroid doses can promote bacterial and fungal superinfections [39].

Idiopathic LPR could be a primary disease, although often accompanies neoplastic and fungal rhinitis [1, 40]. Therefore, it is worth considering that if the dogs’ condition worsens despite therapy re-evaluation is indicated [1, 40]. Moreover inadequate biopsy size or sampling outside the region of neoplastic disease may preclude an accurate and definitive diagnosis [41].

Additionally, three dogs in present study were diagnosed with polyps in rhinoscopy examination. These findings were confirmed by histopathology as an inflammatory with the presence of lymphocytes and plasmocytes. We suspect that these particular three cases, should be precisely diagnosed as polypoid LPR. The resolution of these lesions, and its endoscopic presentation as a polypoid oedema of nasal folds, that is seen in human chronic rhinosinusitis (CRS) [42], may support this statement. In the literature, polyps or polypoid LPR are considered as very rare in dogs [43, 44].

The present study has some potential limitations. Serologic testing is not considered as a good screening test in diagnosis of SNA, as we already mentioned [28]. However, according to previous studies confirmation of fungal disease required at least three positive ancillary diagnostic tests (radiographic, endoscopic, mycologic, histopathologic) [10]. Advanced imaging modalities such as CT or MRI were not performed in this study, thus the differential diagnosis of LPR may be incomplete. However, the current costs of these in Poland still limit their routine use. Moreover, MRI scans or CT findings should always be interpreted in the light of rhinoscopy and histopathology [41]. Histopathology should correlate with clinical suspicion based on nasal CT or repeated biopsies should be performed [41].

In addition, the inspection of the nasal cavities in dogs smaller than 10 kg by the anterograde approach with a rigid endoscope (diameter 2,7 mm) was often incomplete due to the impossibility of performing some movements mandatory for the visualisation of the meatus. Although, the use of the two endoscopic approaches decreased the range of unexaminable areas [2, 45]. It is important to note that other imaging techniques such as CT and MRI are more sensitive than rhinoscopy [45]. However, the rhinoscopy examination allows tissue sampling [45]. On the other hand, endoscopy is a subjective examination and highly depends on the experience of endoscopist [46].

In present study, clinical, endoscopic and histopathologic scoring systems were created to evaluate the efficacy of treatments. The authors are aware of the limitations of such evaluation, which is subjective, but findings were analysed by a clinicist, endoscopist and histopathologist experienced in nasal diseases assessment. Furthermore, the proposed scoring systems were performed according to these previously described in similar studies. Moreover, scoring systems may suggest a method to clinically, endoscopically, or histologically classify rhinitis [10].

Other limitations of this study include small number of cases. Further studies on a larger population are required to validate our findings. Although these findings are very promising, other studies are needed to prove this specific combination therapy as an empirically validated treatment for LPR in dogs.

Furthermore, additional studies are warranted to evaluate the effects of therapies after longer period of time than 6 months, because it is possible that signs of LPR could recur or adverse events presents.

## Conclusions

In conclusion, investigation of the efficacy of three different therapeutic protocols for LPR was performed. The treatment, which involved initially a non-steroidal anti-inflammatory drug, followed by a glucocorticoid appeared to be the most effective in resolving the features of LPR. These patients remained asymptomatic for 6 months.

## Abbreviations

COX-2: Cyclooxygenase- 2
CRS: Chronic Rhinosinusitis
CT: Computed tomography
ELISA: Enzyme-linked immunosorbent assay
H2: Histamine-2 antagonist
IBD: Inflammatory Bowel Disease
LPR: Lymphoplasmacytic rhinitis
MRI: Magnetic Resonance Imaging
NSAID: Non-steroidal anti-inflammatory drug
PPIs: Proton pump inhibitors
SAID: Steroidal anti-inflammatory drug
SNA: Sino-nasal aspergillosis

## References

[1] Meler E, Dunn M, Lecuyer M (2008). A retrospective study of canine persistent nasal disease: 80 cases (1998-2003). Can Vet J.

[2] Windsor RC, Johnson LR (2006). Canine chronic inflammatory rhinitis. Clin Tech Small Anim Pract.

[3] Lobetti RG (2009). A retrospective study of chronic nasal disease in 75 dogs. J S Afr Vet Assoc.

[4] Lobetti RG (2014). Idiopathic lymphoplasmacytic rhinitis in 33 dogs. J S Afr Vet Assoc.

[5] Plickert HD, Tichy A, Hirt RA (2014). Characteristics of canine nasal discharge related to intranasal diseases: a retrospective study of 105 cases. J Small Anim Pract.

[6] Mercier E, Peeters IR, Billen F, Battaille G, Clercx C, Day MJ, Peeters D (2013). Potential role of Alternaria and Cladosporium species in canine lymphoplasmacytic rhinitis. J Small Anim Pract.

[7] Windsor RC, Johnson LR, Herrgesell EJ, De Cock HE (2004). Idiopathic lymphoplasmacytic rhinitis in dogs: 37 cases (1997-2002). J Am Vet Med Assoc.

[8] Tasker S, Knottenbelt CM, Munro EA, Stonehewer J, Simpson JW, Mackin AJ (1999). Aetiology and diagnosis of persistent nasal disease in the dog: a retrospective study of 42 cases. J Small Anim Pract.

[9] Codner EC, Lurus AG, Miller JB, Gavin PR, Gallina A, Barbee DD (1993). Comparison of computed tomography with radiography as a noninvasive diagnostic technique for chronic nasal disease in dogs. J Am Vet Med Assoc.

[10] Furtado ARR, Caine A, Herrtage M (2014). Diagnostic value of MRI in dogs with inflammatory nasal disease. J Small Anim Pract.

[11] Van Pelt DR, McKiernan BC (1994). Pathogenesis and treatment of canine rhinitis. Vet Clin North Am.

[12] Burgener DC, Slocombe RF, Zerbe CA (1987). Lymphoplasmacytic rhinitis in five dogs. J Am Anim Hosp Assoc.

[13] Stepaniuk KS, Gingerich W (2015). Suspect odontogenic infection etiology for canine Lymphoplasmacytic rhinitis. J Vet Dent.

[14] Windsor RC, Johnson LR, Sykes JE, Drazenovich TL, Leutenegger CM, De Cock HEV (2006). Molecular detection of microbes in nasal tissue of dogs with idiopathic Lymphoplasmacytic rhinitis. J Vet Intern Med.

[15] Peeters D (2007). Distinct tissue cytokine and chemokine mRNA expression in canine sino-nasal aspergillosis and idiopathic lymphoplasmacytic rhinitis. Vet Immunol Immunopathol.

[16] KuKanich B, Bidgood T, Knesl O (2012). Clinical pharmacology of nonsteroidal anti- inflammatory drugs in dogs. Vet Anesth Anal.

[17] Lascelles BDX, McFarland JM, Swann H (2005). Guidelines for safe and effective use of NSAIDs in dogs. Vet Therap.

[18] Viviano KR. A practical approach to immunosuppressive therapies; AAHA/OVMA Toronto 2011 Proceedings; 2011 Mar 24-27; Toronto. Lakewood: American Animal Hospital Association; 2011.

[19] Vivano KR (2013). Update on Immunosupressive therapies for dogs and cats. Vet Clin Small Anim.

[20] Daure E, Ross L, Webster CR (2017). Gastroduodenal ulceration in small animal: part 2. Proton pump inhibitors and histamine- 2 receptor antagonists. J Am Med Hosp Assoc.

[21] Neiger R (2003). Editorial: NSAID- induced gastrointestinal adverse effects in dogs- can we avoid them?. J Vet Intern Med.

[22] Willard MD, Silverstein DC, Hopper K (2009). Gastrointestinal Protectants. Small Animal Critical Care Medicine.

[23] Gwee KA, Goh V, Lima G, Setia S (2018). Coprescribing proton- pump inhibitors with nonsteroidal anti- inflammatory drugs: risks versus benefits. J Pain Res.

[24] Henderson AK, Webster CRL. The use of Gastroprotectants in treating gastric ulceration in dogs. CE. 2006;28(5):358–70.

[25] Bersenas AME, Mathews KA, Allen DG, Conlon PD (2005). Effects of ranitidine, famotidine, pantoprazole, and omeprazole on intragastric pH in dogs. AJVR.

[26] Johnson LR, Clarke HE, Bannasch MJ, De Cock HE (2004). Correlation of rhinoscopic signs of inflammation with histologic findings in nasal biopsy specimens of cats with or without upper respiratory tract disease. J Am Vet Med Assoc.

[27] Furtado ARR, Constantino- Casas F (2013). Histopathology inflammation scoring and classification in 34 dogs with inflammatory nasal disease. Vet Rec.

[28] Billen F, Peeters D. Aspergillosis- canine. In: Ettinger SJ, Feldman EC, Cote E, editors. Textbook of Veterinary Internal Medicine 1035- 1039. 2017.

[29] Lodrup AB, Reimer C, Bytzer P (2013). Systematic review: symptoms of rebound acid hypersecretion following proton pump inhibitor treatment. Scand J Gastroenterol.

[30] Mordecai A, Sellon RK, Mealey KL (2011). Normal dogs treated with famotidine for 14 days have only transient increases in serum gastrin concentrations. J Vet Intern Med.

[31] Suleyman H, Demircan B, Karagoz Y (2007). Anti- inflammatory and side effects of cyclooxygenase inhibitors. Pharmacol Rep.

[32] Distel M, Mueller C, Bluhmki E, Fries J (1996). Safety of meloxicam: a global analysis of clinical trials. Br J Rheumatol.

[33] Luna Stelio P. L., Basílio Ana C., Steagall Paulo V. M., Machado Luciana P., Moutinho Flávia Q., Takahira Regina K., Brandão Cláudia V. S. (2007). Evaluation of adverse effects of long-term oral administration of carprofen, etodolac, flunixin meglumine, ketoprofen, and meloxicam in dogs. American Journal of Veterinary Research.

[34] Maeda Y, Tanaka R, Ohtsuka H, Matsuda K, Tanabe T, Oikawa M (2011). Comparison of the immunosuppressive effects of dexamethasone, Flunixin, Meglumine and meloxicam on the in vitro response of calf peripheral blood mononuclear cells. J Vet Med Sci.

[35] Chacon P, Vega A (2005). Induction of cyclooxygenase-2 expression by allergens in lymphocytes from allergic patients. Eur J Immunol.

[36] Iniquez MA, Punzon C, Fresno M (1999). Induction of cyclooxygenase- 2 on activated T lymphocytes regulation of T cell activation by cyclooxygenase- 2 inhibitors. J Immunol.

[37] Jergens AE, Crandell J (2010). Comparison of Oral prednisone and prednisone combined with metronidazole for induction therapy of canine inflammatory bowel disease: a randomized- controlled trial. J Vet Inten Med.

[38] Lappin MR, Blondeau J, Boothe EB (2017). Antimicrobial use guidelines for treatment of respiratory tract disease in dogs and cats: antimicrobial Guideliness working Group of the International Society for companion animal infectious diseases. J Vet Intern Med.

[39] Sequin MA, Vaden SL, Altier C, Stone E, Levine JF (2003). Persistent urinary tract infections and reinfections in 100 dogs (1989-1999). J Vet Intern Med.

[40] Gieger T, Northrup N. Clinical approach to patients with epistaxis. CE. 2004;26(1):30–42.

[41] Lefebvre J, Kuehn NF, Wortinger A (2005). Computed tomography as an aid in the diagnosis of chronic nasal disease in dogs. J Small Anim Pract.

[42] Snidvongs K, Dalgorf D, Kalish L, Sacks R, Pratt E, Harvey RJ (2013). Modified Lund Mackay postoperative endoscopy score for defining inflammatory burden in chronic rhinosinusitis. Rhinology.

[43] Holt DE, Goldschmidt MH (2011). Nasal polyps in dogs: five cases. J Small Anim Practic.

[44] Labuc R. The Approach to Nasal Discharge in the Dog: The 8^th^ Annual vet education online Vetrinary conference; 2017. Vet Education Pty Ltd 2017. https://webinars.veteducation.com.au/wpcontent/images/Online-Conference-2017-Nasal-Disease-Lecture-Notes.pdf.

[45] Pietra M, Spinella G, Pasquali F, Romagnoli N, Bettini G, Spadari A (2010). Clinical findings, rhinoscopy and histological evaluation of 54 dogs with chronic nasal disease. J Vet Sci.

[46] Soliz- Munos P (2014). Experience of the endoscopist increases detection rates of smaller size and higher histological grade polyps. J Gastroenterol Hepatol.

